# Whole blood angiopoietin-1 and -2 levels discriminate cerebral and severe (non-cerebral) malaria from uncomplicated malaria

**DOI:** 10.1186/1475-2875-8-295

**Published:** 2009-12-15

**Authors:** Andrea L Conroy, Erin I Lafferty, Fiona E Lovegrove, Srivicha Krudsood, Noppadon Tangpukdee, W Conrad Liles, Kevin C Kain

**Affiliations:** 1 Sandra A. Rotman Laboratories, McLaughlin-Rotman Centre for Global Health, University Health Network-Toronto General Hospital, McLaughlin Centre for Molecular Medicine, University of Toronto, Toronto ON, Canada; 2 Faculty of Tropical Medicine, Mahidol University, Bangkok, Thailand; 3 Tropical Disease Unit, Division of Infectious Diseases, Department of Medicine, University of Toronto, Toronto ON, Canada

## Abstract

**Background:**

Severe and cerebral malaria are associated with endothelial activation. Angiopoietin-1 (ANG-1) and angiopoietin-2 (ANG-2) are major regulators of endothelial activation and integrity. The aim of this study was to investigate the clinical utility of whole blood angiopoietin (ANG) levels as biomarkers of disease severity in *Plasmodium falciparum *malaria.

**Methods:**

The utility of whole blood ANG levels was examined in Thai patients to distinguish cerebral (CM; n = 87) and severe (non-cerebral) malaria (SM; n = 36) from uncomplicated malaria (UM; n = 70). Comparative statistics are reported using a non-parametric univariate analysis (Kruskal-Wallis test or Chi-squared test, as appropriate). Multivariate binary logistic regression was used to examine differences in whole blood protein levels between groups (UM, SM, CM), adjusting for differences due to ethnicity, age, parasitaemia and sex. Receiver operating characteristic curve analysis was used to assess the diagnostic accuracy of the ANGs in their ability to distinguish between UM, SM and CM. Cumulative organ injury scores were obtained for patients with severe disease based on the presence of acute renal failure, jaundice, severe anaemia, circulatory collapse or coma.

**Results:**

ANG-1 and ANG-2 were readily detectable in whole blood. Compared to UM there were significant decreases in ANG-1 (p < 0.001) and significant increases in ANG-2 (p < 0.001) levels and the ratio of ANG-2: ANG-1 (p < 0.001) observed in patients with SM and CM. This effect was independent of covariates (ethnicity, age, parasitaemia, sex). Further, there was a significant decrease in ANG-1 levels in patients with SM (non-cerebral) versus CM (p < 0.001). In participants with severe disease, ANG-2, but not ANG-1, levels correlated with cumulative organ injury scores; however, ANG-1 correlated with the presence of renal dysfunction and coma. Receiver operating characteristic curve analysis demonstrated that the level of ANG-1, the level of ANG-2 or the ratio of ANG-2: ANG-1 discriminated between individuals with UM and SM (area under the curve, p-value: ANG-2, 0.763, p < 0.001; ANG-1, 0.884, p < 0.001; Ratio, 0.857, p < 0.001) or UM and CM (area under the curve, p-value: ANG-2, 0.772, p < 0.001; ANG-1, 0.778, p < 0.001; Ratio, 0.820, p < 0.001).

**Conclusions:**

These results suggest that whole blood ANG-1/2 levels are promising clinically informative biomarkers of disease severity in malarial syndromes.

## Background

Only a small proportion of individuals with *Plasmodium falciparum *malaria progress to severe and potentially fatal forms of infection [1,2]. The definitive diagnosis of severe and cerebral malaria is challenging due to the non-specific nature of the clinical presentation and the confounder of incidental parasitaemia in malaria-endemic areas [3]. These factors may result in misdiagnosis and adverse outcomes due to the failure to treat other life-threatening infections [2,3]. In studies of African children diagnosed with cerebral malaria, over 20% were shown to have an alternative cause for their neurological syndrome at post-mortem examination [3]. A rapid point-of-care test that accurately identifies patients with severe or cerebral malaria, or those at risk of progressing to these syndromes, would be of clinical and public health utility. However, limited prognostic or diagnostic laboratory tools for severe malaria are currently available.

Endothelial activation and dysfunction have been implicated in the pathogenesis of severe and cerebral malaria [4-11]. The angiogenic factors, angiopoietin-1 (ANG-1) and angiopoietin-2 (ANG-2) have recently been shown to function as essential regulators of endothelial activation and integrity [12]. ANG-1 is constitutively expressed and maintains vascular quiescence by signaling though the Tie-2 receptor [12]. ANG-2 is released from Weibel-Palade (WP) bodies in association with endothelial activation and displaces ANG-1; sensitizing the endothelium to become responsive to sub-threshold concentrations of cytokines such as TNF [12]. Elevations in plasma or serum ANG-2 levels have been reported in patients with sepsis, acute lung injury/acute respiratory distress syndrome [13,14], and severe malaria [8,11].

Based on the hypothesis that dysregulation of angiopoietins is associated with severe malaria syndromes, ANG-1 and ANG-2 levels were examined in malaria-infected patients to determine if they would distinguish between uncomplicated, severe and cerebral malaria. This study focused on the predictive value of ANG-1 and ANG-2 levels in whole blood, since unprocessed whole blood obtained by finger prick is a preferred clinical specimen for point-of-care testing [15].

## Methods

### Study population and specimen collection

Individuals (≥ 13 years of age) presenting with falciparum malaria to Hospital for Tropical Disease clinics (Mahidol University, Thailand) were eligible for enrolment into this study. Patients were classified into uncomplicated (UM), cerebral (CM) or severe (non-cerebral) malaria (SM) according to WHO criteria [2]. Whole blood samples (pre-treatment) were collected from all patients for thick and thin blood film preparation, PCR, and complete blood counts. A heparinized aliquot was frozen at -80°C for determination of subsequent angiopoietin levels. An expert microscopist, who was blinded to the results of additional diagnostic testing, examined the blood films. Smears were considered negative if no parasites were seen in two consecutive thick blood films. Parasite density was calculated by thick or thin film determining the number of parasites per 200 white blood cells for thick blood films or per 1,000 red blood cells (RBC) for thin blood films. Baseline white blood cell counts or RBC counts were used to calculate parasitaemia (parasites per μL). *Plasmodium falciparum *diagnosis was confirmed by PCR as previously described [16]. The institutional review board of the Faculty of Tropical Medicine, Mahidol University approved this study and informed consent was obtained from all patients or their legal guardians prior to specimen collection.

### Quantification of ANG-1 and ANG-2 in whole blood

Whole blood concentrations of ANG-1 and ANG-2 were measured by ELISA (R&D Systems, Minneapolis MN) according to the manufacturers' instructions. Concentrations were interpolated from 4-parameter-fit standard curves generated using a standard curve of recombinant human angiopoietin proteins. The lower and upper limits of detection for each assay were as follows: ANG-1 (156.25 - 10,000 pg/mL) and ANG-2 (54.69 - 3,500 pg/mL).

### Statistical analyses

Statistical analysis was performed using SPSS v17.0. Comparative statistics are reported from a non-parametric univariate analysis (Kruskal-Wallis test, followed by Mann-Whitney U test with adjustment for multiple comparisons or Chi-squared test, as appropriate). Multivariate binary logistic regression was used to examine differences in whole blood protein levels between groups (UM, SM, CM), adjusting for differences due to ethnicity, age, parasitaemia and sex. Spearman's correlation was used to examine correlations between angiopoietin levels and parasitaemia or cumulative organ injury scores. Receiver operating characteristic (ROC) curves and area under the ROC curves were generated using SPSS v.17.0. Optimal test thresholds were derived mathematically from the ROC curves. To adjust for multiple comparisons at a family-wise simultaneous error rate of α = 0.05, Bonferonni corrections were applied as appropriate.

## Results

### Study population

193 patients with *P. falciparum *malaria were enrolled during the study. Patients were classified as having UM, (n = 70), SM (n = 36) or CM (n = 87) [2]. In patients with complicated disease (SM and CM), 22 participants (17.9%) had evidence of acute renal failure, 71 had evidence of liver dysfunction (57.7%), three had algid malaria with a systolic blood pressure less than 80 mmHg (2.4%), 34 were anaemic and required a blood transfusion (27.6%) and 87 were in a coma (70.7%). Of study participants with severe malaria, 46 had a single organ system involved, 41 had two organ systems affected, 22 had three and five patients had involvement of four organ systems. There was a single fatality in the study with the study participant having cerebral malaria and algid malaria. Demographic and baseline clinical characteristics of the study participants are shown in Table 1.

**Table 1:** Demographic characteristics of Thai adults with malaria

	Uncomplicated Malaria	Severe Malaria (Non-cerebral)	Severe Malaria (Cerebral)	P
**Sample size**	70	36	87	

**Sex, Number (%)**				

**Male**	46 (80)	27 (75)	73 (84)	

**Female**	14 (20)	9 (25)	14 (16)	0.508^a^

**Age, years**	26 (13-50)	24 (14-59)	26 (14-61)	0.184 ^b^

**Parasitaemia, Parasites/uL**	5360 (422385)	119850 (943890)	84840 (1188626)	< 0.0001^b^

**NOTE**: Data are median (range) unless otherwise indicated.

^a ^Chi-squared test.

^b ^Kruskall-Wallis U test.

### Whole blood ANG-2 levels are increased and ANG-1 levels are decreased in SM or CM compared to UM

**Figure 1:**
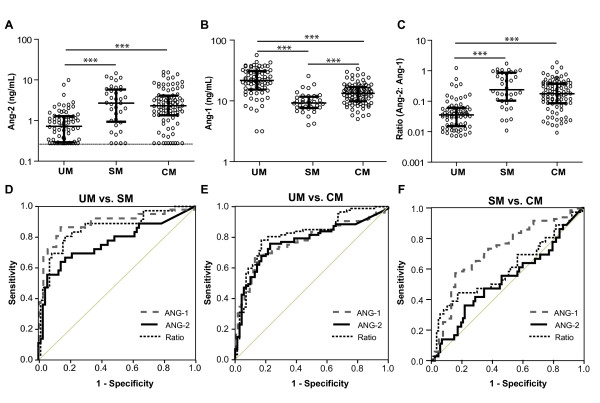
**Comparison of angiopoietin-1 and -2 levels in whole blood samples from Thai adults with malaria**. **A-C**. Whole blood concentrations of (A) angiopoietin-2 (ANG-2), (B) angiopoietin-1 (ANG-1), and (C) the ratio of ANG-2:ANG-1 (Ratio, expressed as log base 10) were measured in 70 uncomplicated malaria (UM) patients, 36 severe malaria (SM) patients, and 87 cerebral malaria (CM) patients. ***p < 0.0001 by Kruskal-Wallis test with posthoc comparisons using Mann-Whitney U test and Bonferonni correction. **D-F**. Receiver operating characteristic (ROC) curves were generated for each test to compare (D) UM with SM patients, (E) UM with CM patients, and (F) SM with CM patients, with the null hypothesis (diagonal line) that the area under the curve equals 0.5.

Whole blood ANG-2 levels were significantly higher in individuals with SM or CM compared to individuals with UM (Figure 1A; p < 0.001). Furthermore, ANG-1 levels were significantly lower in those with SM compared to individuals with either UM or CM, and in individuals with CM compared to UM (Figure 1B; p < 0.001). Since ANG-2 and ANG-1 have divergent roles in vascular activation, the ratio of ANG-2:ANG-1 was used as an additional measure for each patient. This ratio was significantly higher in patients with SM or CM compared to those with UM (Figure 1C; p < 0.001).

### Receiver operating characteristic (ROC) curves indicate that angiopoietin levels discriminate between UM and SM or CM

ROC curves were plotted for ANG-2, ANG-1, and the ratio of ANG-2:ANG-1 to assess the ability of each biomarker to discriminate between UM, SM and CM. Comparing individuals with UM vs. individuals with SM, all markers have an area under the curve (AUC) that differs significantly from that of a chance result (AUC: 0.5) (Figure 1D: AUC, p-value: ANG-2, 0.763, p < 0.001; ANG-1, 0.884, p < 0.001; Ratio, 0.857, p < 0.001). Similarly, all markers discriminated between UM and CM (Figure 1E: AUC, p-value: ANG-2, 0.772, p < 0.001; ANG-1, 0.778, p < 0.001; Ratio, 0.820, p < 0.001). Finally, ANG-1 but not ANG-2 or the ANG-2:ANG-1 ratio, was able to discriminate between SM and CM (Figure 1F: AUC, p-value: ANG-1; 0.735, p < 0.001: ANG-2; 0.527, p = 0.663: Ratio; 0.599, p = 0.084) with ANG-1 levels being lower in SM than CM. In this population, ANG-1 discriminated between UM and severe malarial syndromes better than either ANG-2 or the ratio of ANG-2:ANG-1, and was able to distinguish SM from CM.

### The ratio of ANG-2:ANG-1 displays high sensitivity and specificity as a biomarker for disease severity

The sensitivity, specificity, and positive and negative likelihood ratios (LR(±)) were determined for ANG-1, ANG-2, and the ratio of ANG-2:ANG-1. Based on sensitivity and specificity, ANG-1 was best able to discriminate between UM and SM (cut-off (12.38 ng/mL): sensitivity: 0.861, specificity: 0.857, LR(+): 6.028, LR(-): 0.162)); whereas ANG-2 was better at discriminating between UM and CM (cut-off (1.33 ng/mL): sensitivity: 0.759, specificity: 0.771, LR(+): 3.319, LR(-): 0.313)). The ratio of ANG-2:ANG-1 was best at discriminating between complicated disease of mixed phenotype (SM+CM) (cut-off 0.08): sensitivity: 0.789, specificity: 0.829, LR(+): 4.60, LR(-): 0.264)) (Table 2). Collectively, our data suggest that the ratio of ANG-2:ANG-1 may be the best predictor of patients with uncomplicated disease versus patients with complicated (severe or cerebral) disease, whereas ANG-1 may have utility in differentiating between cerebral malaria vs. severe (non-cerebral) disease.

**Table 2:** Optimal biomarker cut-off values for receiver operating characteristic curve sensitivity, specificity, positive likelihood ratio (LR(+)) and negative likelihood ratio (LR(-)) at the chosen cutoffs.

	Cut-off	Sensitivity	Specificity	LR(+)	LR(-)
Angiopoietin-2	ng/mL	# (95% CI)	# (95% CI)	# (95% CI)	# (95% CI)

UM vs. SM	1.43	0.694 (0.531-0.820)	0.786 (0.676-0.866)	3.241 (1.969-5.333)	0.389 (0.234-0.646)
UM vs. CM	1.33	0.759 (0.659-0.836)	0.771 (0.661-0.854)	3.319 (2.124-5.186)	0.313 (0.211-0.464)
UM vs. (CM+SM)	1.33	0.740 (0.656-0.809)	0.771 (0.661-0.854)	3.237 (2.078-5.041)	0.337 (0.244-0.466)
SM vs. CM	3.14	0.472 (0.319-0.630)	0.644 (0.539-0.736)	1.325 (0.848-2.070)	0.820 (0.580-1.159)

Angiopoietin-1	ng/mL	# (95% CI)	# (95% CI)	# (95% CI)	# (95% CI)

UM vs. SM	12.38	0.861 (0.713-0.939)	0.852 (0.757-0.921)	6.028 (3.346- 10.859)	0.162 (0.071-0.368)
UM vs. CM	15.86	0.713 (0.610-0.797)	0.743 (0.630-0.831)	2.771 (1.821-4.218)	0.387 (0.270-0.554)
UM vs. (CM+SM)	15.16	0.707 (0.622-0.781)	0.771 (0.661-0.854)	3.094 (1.983-4.830)	0.379 (0.280-0.514)
SM vs. CM	11.21	0.722 (0.560-0.842)	0.655 (0.551-0.747)	2.094 (1.471-2.982)	0.424 (0.245-0.734)

Ratio		# (95% CI)	# (95% CI)	# (95% CI)	# (95% CI)

UM vs. SM	0.082	0.806 (0.650-0.903)	0.829 (0.724-0.899)	4.699 (2.74-8.059)	0.235 (0.120-0.460)
UM vs. CM	0.080	0.782 (0.684-0.856)	0.829 (0.724-0.899)	4.559 (2.692-7.722)	0.264 (0.175-0.398)
UM vs. (CM+SM)	0.080	0.789 (0.708-0.852)	0.829 (0.723-0.899)	4.600 (2.727-7.762)	0.255 (0.178-0.365)
SM vs. CM	0.448	0.444 (0.295-0.604)	0.828 (0.735-0.893)	2.578 (1.432-4.639)	0.671 (0.494-0.913)

### Angiopoietins predict severe malaria independently of covariates

In order to examine whether endothelial activation was independent of parasite burden, levels of ANG-1 and ANG-2 were correlated with parasitaemia. Overall, there was a positive correlation between ANG-2 and parasitaemia (Spearman's rho 0.339, p < 0.0001) and a negative correlation between ANG-1 and parasitaemia (Spearman's rho -0.446, p < 0.0001). Furthermore, ANG-2 and ANG-1 were negatively correlated with one another (Spearman's rho -.320, p < 0.0001). Multivariate logistic regression analysis revealed that the observed differences in ANG levels between groups remained even after adjusting for differences due to ethnicity, age, gender, and parasitaemia. There remained a significant increase in ANG-2 (and the ratio of ANG-2:ANG-1) in SM vs. UM, and CM vs. UM, with no significant difference between SM and CM. For ANG-1, there was a significant decrease in SM vs. UM, CM vs. UM, and SM vs. CM (p < 0.0001).

### Angiopoietin-2 is associated with cumulative organ injury

To address whether ANG levels are a measure of disease severity, an organ injury score ranging from 0-5 was assigned to each participant based on the number of organ systems involved. A value of 1 was added to the organ injury score for each participant based on the presence of each of the following: acute renal failure requiring haemodialysis, clinically apparent jaundice, severe anaemia requiring a blood transfusion, circulatory collapse with a systolic pressure <80 mmHg, and coma. ANG-2 and the ratio of ANG-2: ANG-1 were positively correlated with cumulative organ injury (Spearman's rho, p-value: ANG-2; 0.378, p < 0.0001: ANG-2:ANG-1 ratio; 0.288, p = 0.001). Interestingly, ANG-1 also trended towards a positive correlation (Spearman's rho 0.166, p = 0.071) despite its ability to discriminate between individuals with cerebral vs. severe non-cerebral malaria. To investigate this further, a post-hoc analysis was performed to examine how ANG-1 correlated with the organ systems included in the analysis. There were no associations observed between ANG-1 levels in participants with hepatic dysfunction, severe anaemia or circulatory collapse, although the latter was underpowered. However, ANG-1 was positively associated with renal dysfunction (Spearman's rho, p-value: 0.243, p = 0.007) and coma (Spearman's rho, p-value: 0.370, p < 0.0001).

## Discussion

These results demonstrate that whole blood ANG-1 and ANG-2 levels are robust biomarkers of severe and cerebral malaria. ANG-2, ANG-1 and the ratio of ANG-2: ANG-1 were significantly different between UM and both SM and CM (Figure 1A-C). Of particular interest, ANG-1 levels further distinguished patients with severe (non-cerebral) malaria from those with cerebral involvement (Figure 1B). Finally, ROC curve analysis indicated good diagnostic accuracy for these biomarkers in discriminating uncomplicated malaria from severe malarial syndromes (Figure 1D-F; Table 2). Significant differences between groups remained after correcting for potential confounding factors, and were independent of parasitaemia, ethnicity, age and gender. Taken together with recent studies, these data implicate dysregulation of angiopoietins in the pathogenesis of complicated malaria and suggest they may be clinically informative biomarkers of disease severity [8,11].

The ability of angiopoietins to discriminate between uncomplicated and complicated malaria has now been confirmed in three distinct geographic regions and populations (children in Uganda, adults in Papua and Thailand). These proteins have previously been measured in serum (ANG-1, ANG-2) [8], plasma (ANG-2) [11], and here we show that they can be measured directly in whole blood samples acquired from an independent population of malaria-infected individuals. Only a small number of patients progress to severe and fatal malaria [1,2] and a rapid point-of-care test to identify malaria-infected individuals with or at risk of progressing to severe disease could be of clinical utility. ANG-1 and ANG-2 are attractive candidates for incorporation into rapid lateral flow immunochromatographic tests combined with malaria antigen detection due to their detection in whole blood and ability to discriminate between patients with and without severe disease. However, before biomarkers like ANG-1 or ANG-2 can be incorporated into rapid malaria tests and clinical practice, additional prospective studies will be needed to confirm and validate their usefulness in different populations. Optimal cut-offs need to be determined using standardized methods of sample collection, processing, and laboratory protocols for biomarker testing.

ANG-1 and ANG-2 are stable proteins able to withstand repeated freeze thaws without significant changes in protein levels, and are readily detectable, as this study demonstrates, in whole blood. Furthermore, the angiopoietins, which are linked with inflammation, angiogenesis, and endothelial function and integrity, play a central role in many of the disease processes implicated in the pathogenesis of severe malarial syndromes. ANG-2 exists pre-stored within WP bodies and is rapidly released upon, and may further contribute to, endothelial activation [12]. As a result, changes in ANG-2 levels may represent early changes in the endothelial beds, and future studies should examine its utility as a predictive biomarker. Furthermore, different endothelial beds may be differentially responsive to exogenous stimuli, providing a putative mechanism to explain why certain syndromes of organ dysfunction are more common in severe malaria, particularly acute renal injury, respiratory distress, or cerebral malaria. This may be particularly relevant with ANG-1, which differentiated between severe (non-cerebral) malaria and cerebral malaria in this study.

In the current study, ANG-2 levels were elevated in severe malarial syndromes compared to uncomplicated malaria but were also correlated with scores of cumulative organ injury in the cohort of severe malaria patients. This confirms other studies suggesting that ANG-2 is a marker of disease severity in conditions associated with endothelial activation [8,11,13,14]. However, this is the first study to show that ANG-1 differentiates between severe (non-cerebral) and cerebral malaria. Interestingly, ANG-1 levels did not correlate significantly with the cumulative organ injury score but were specifically correlated with renal dysfunction and coma. It is unclear whether the association between ANG-1 levels and the presence of renal impairment and coma are due to the nature of microvascular environment in those organ systems or other physiological derangements. In order to address those questions, these observations will need to be confirmed in a larger population of individuals with well-characterized clinical phenotypes.

The use of biomarkers is inherently limited by their specificity for the disease in question. Dysregulation of angiopoietins may occur in a number of severe infectious syndromes associated with endothelial activation and dysfunction. As a result, it will be important to consider the implications of co-infections, including bacterial and viral (e.g. HIV) infections if these proteins are to be used as prognostic indicators for malaria.

Finally, based on our study design it was not possible to examine the predictive value of angiopoietins and future prospective studies are needed to examine the kinetics of angiopoietin levels and their relation to symptom onset, clinical history of malaria, and response to treatment. However, should angiopoietins fail to distinguish between malaria and other infectious diseases, these proteins may still be informative biomarkers to facilitate clinical decision-making (e.g. patient triage, referral, admission) and optimal allocation of health resources for the treatment of severe infections associated with endothelial dysfunction.

## Conclusions

In summary, these data suggest that ANG-1 and ANG-2 are promising biomarkers of severe malaria. Furthermore, the ability to robustly detect angiopoietin levels in whole blood makes them attractive candidates for potential integration into point-of-care diagnostic/theranostic devices of disease severity.

## Competing interests

The University Health Network holds intellectual property related to the role of angiogenic factors in the pathogenesis of infectious disease. The authors declare that they have no competing interests.

## Authors' contributions

ALC performed data analysis and drafted the manuscript. FEL contributed to the study design and performed the experiments. EIL performed the experiments. NT and SK contributed to the study design and collection of patient samples. WCL and KCK conceived of the idea, contributed to study design and helped write the manuscript. All authors read and approved the final manuscript.
